# Abbreviated Exposure to Hypoxia Is Sufficient to Induce CNS Dysmyelination, Modulate Spinal Motor Neuron Composition, and Impair Motor Development in Neonatal Mice

**DOI:** 10.1371/journal.pone.0128007

**Published:** 2015-05-28

**Authors:** Jens O. Watzlawik, Robert J. Kahoud, Ryan J. O’Toole, Katherine A. M. White, Alyssa R. Ogden, Meghan M. Painter, Bharath Wootla, Louisa M. Papke, Aleksandar Denic, Jill M. Weimer, William A. Carey, Moses Rodriguez

**Affiliations:** 1 Department of Neurology, Mayo Clinic College of Medicine, Rochester, Minnesota, United States of America; 2 Department of Pediatric and Adolescent Medicine, Mayo Clinic College of Medicine, Rochester, Minnesota, United States of America; 3 Children's Health Research Center, Sanford Research, Sioux Falls, South Dakota, United States of America; Massachusetts General Hospital/Harvard Medical School, UNITED STATES

## Abstract

Neonatal white matter injury (nWMI) is an increasingly common cause of cerebral palsy that results predominantly from hypoxic injury to progenitor cells including those of the oligodendrocyte lineage. Existing mouse models of nWMI utilize prolonged periods of hypoxia during the neonatal period, require complex cross-fostering and exhibit poor growth and high mortality rates. Abnormal CNS myelin composition serves as the major explanation for persistent neuro-motor deficits. Here we developed a simplified model of nWMI with low mortality rates and improved growth without cross-fostering. Neonatal mice are exposed to low oxygen from postnatal day (P) 3 to P7, which roughly corresponds to the period of human brain development between gestational weeks 32 and 36. CNS hypomyelination is detectable for 2–3 weeks post injury and strongly correlates with levels of body and brain weight loss. Immediately following hypoxia treatment, cell death was evident in multiple brain regions, most notably in superficial and deep cortical layers as well as the subventricular zone progenitor compartment. PDGFαR, Nkx2.2, and Olig2 positive oligodendrocyte progenitor cell were significantly reduced until postnatal day 27. In addition to CNS dysmyelination we identified a novel pathological marker for adult hypoxic animals that strongly correlates with life-long neuro-motor deficits. Mice reared under hypoxia reveal an abnormal spinal neuron composition with increased small and medium diameter axons and decreased large diameter axons in thoracic lateral and anterior funiculi. Differences were particularly pronounced in white matter motor tracts left and right of the anterior median fissure. Our findings suggest that 4 days of exposure to hypoxia are sufficient to induce experimental nWMI in CD1 mice, thus providing a model to test new therapeutics. Pathological hallmarks of this model include early cell death, decreased OPCs and hypomyelination in early postnatal life, followed by dysmyelination, abnormal spinal neuron composition, and neuro-motor deficits in adulthood.

## Introduction

The rate of cerebral palsy (CP) has increased steadily over the past few decades to its current incidence of more than 3 per 1000 live births [1]. Much of this increase is attributed to the improved survival of neonates born prematurely and at extremely low birth weight (ELBW) [1]. Well beyond infancy and childhood, those ELBW who survive with CP display motor deficits and cognitive-behavioral disturbances that correlate closely with the neuropathology of neonatal white matter injury (nWMI), formerly known as periventricular leukomalacia (PVL) when associated with cystic necrosis [2–6]. There are no effective drugs or therapeutic strategies currently available to prevent or cure these neurodevelopmental disorders or the underlying neuropathology.

White matter disease predominates in nWMI and manifests as diffuse hypomyelination and reduced white matter volume in the cerebral cortex [4]. At a cellular level, nWMI may at least partially originate from early developmental arrest and/or death of oligodendrocyte progenitor cells (OPCs); [7, 8]. The pool of Olig-2+ OPCs/immature OLs increased post hypoxia in the presence of astrogliotic, necrotic lesions [7, 8] or remained unchanged in noncystic nWMI [9]. Irrespective of the origin of the OL-lineage cell damage, nWMI results in abnormal myelination of mature myelin basic protein (MBP)-positive OLs despite normal total myelin levels in the developed brain [8]. This indicates a qualitative rather than quantitative hypoxia-mediated OL deficit in preterm neonates. Given the sensitivity of the OL lineage to hypoxic stress [10], it is widely accepted that the episodic recurrence of hypoxia-ischemia in ELBW neonates is a leading contributor to alterations in the OL lineage progression and, consequently, nWMI [4, 11–13]. A recent study strongly emphasized the importance of oligodendrocyte-lineage cells in nWMI by demonstrating rescue of the severe neuro-motor phenotype after hypoxia through stimulation of OPC proliferation [14]. In addition to OL-lineage cells Verney et al., (2012) emphasized a potential role of activated microglia in disabilities after brain injury in very preterm infants (gestational weeks 25–29).

Given the link between hypoxic stress and the pathogenesis of nWMI, a number of investigators have modeled the disease in experimental animals by exposing neonatal rodent pups to various degrees and durations of hypoxia [15–21]. In both mice and rats, exposure to 9–11% oxygen for 7 to 30 days during the first month of life yields a spectrum of white matter diseases that closely resembles nWMI seen in human ELBWs. Specifically, hypoxia reduces the volumes of the cerebral cortex, subcortical white matter and the corpus callosum, followed by progressive ventriculomegaly [10, 15, 22, 23].

Whereas these murine models are well established in the literature and valuable for the study of nWMI, they are fraught with limitations. The hypoxic exposure most typically begins at postnatal day (P) 3 and continues for 8 to 11 days [10, 14, 15, 18, 23–26]. This period roughly correlates with human brain development between 32 weeks gestation through the first year of postnatal life [27–30]. Thus, this hypoxia exposure paradigm does not selectively target the early phases of OL development relevant to human nWMI, which typically ends in human term neonates or P7 in rodents [30]. In addition, the timing and duration of this hypoxic exposure reduces skeletal muscle mass, body and brain weights, and overall survival, suggesting that malnourishment and/or systemic illness may confound interpretation in these models [22, 31, 32]. Moreover, attempts to obviate these two confounding factors, such as co- or cross-fostering neonatal mice by dams of another strain [15, 23] add cost and an undesirable degree of complexity to the *in vivo* study of nWMI.

In the present study, we tested whether exposure of neonatal mice to 4 days of hypoxia (abbreviated hypoxia) from P3 to P7 in an outbred CD1 mouse strain is sufficient to induce the neuropathological and functional deficits consistent with human nWMI. We also evaluated how cross-fostering neonatal mice during and after hypoxia impacts survival, growth, and myelination. In addition, we identified pathological changes of myelin and axons that correlate with persistent neuro-motor deficits in our model of nWMI.

## Methods

### Experimental animals

Timed-pregnant CD1 mice were obtained from Charles River Laboratories and maintained in usual conditions. The litter size used until P21 was 12 per dam. All animals were cared for according to all local, state and federal regulations and consistent with protocol approved by the Mayo Clinic Institutional Animal Care and Use Committee (IACUC) and the National Institute of Health. IACUC protocol number: A51912, Title of project: "Models of Neurologic Disease". In order to minimize animal suffering all mice were anesthetized before intracardial perfusion using Pentobarbital.

### Hypoxia-induced white matter disease model

#### Abbreviated Hypoxia

Litters of male and female CD1 mice were randomly assigned for rearing in hypoxia or room air (normoxia) from P3 to P7, using a litter size of 12 for all dams throughout the study. For litters assigned to hypoxic rearing, cages were placed within an acrylic chamber that was ventilated with nitrogen to lower ambient oxygen tension of 10 +/- 0.5%. On P7, these cages were removed from the chamber, and mice were subsequently reared in usual, normoxic conditions. Litters assigned to normoxia were reared in usual conditions throughout the study. Mice from both exposure groups were randomly sacrificed at P13, P27 or P80 for use in the histological and molecular studies described below. At the time of sacrifice, body weights and brain weights were measured. Additional mice were randomly selected and maintained in usual conditions for use in neuromotor testing at P21, P43 and P80.

#### Standard hypoxia

In order to compare our hypoxia model with a well-established model of nWMI, we randomly assigned litters of CD1 mice for rearing in hypoxia or normoxia from P3 to P12 [15–21] (n = 20 for standard hypoxia, n = 102 for abbreviated hypoxia, n = 102 for normoxia, see also Table 1). As above, body weight and brain weights were measured at the time of sacrifice.

**Table 1:** Long hypoxia vs abbreviated hypoxia.

	Abbreviated hypoxia (P3 → P7)	Long hypoxia (P3 → P12)	Normoxia
**Survival rate** (in percent)	100 (102/102) (P12)	35 (7/20) (P12)	100 (102/102)
**Body weight (g)** (mean ± std.-dev.)	5.12 ± 0.08 (P12) (abbreviated hypoxia vs. long hypoxia, (p < 0.001)	2.90 ± 0.30 (P12) (normoxia vs. long hypoxia, p < 0.001)	7.20 ± 0.60 (P12)
**Brain weight** (mean ± std.-dev.)	0.33 ± 0.005 (P12) (abbreviated hypoxia vs. long hypoxia, (p < 0.001)	0.21 ± 0.03 (P12) (normoxia vs. long hypoxia, p < 0.001)	0.39 ± 0.03 (P12)

Oxygen levels were monitored continuously during the exposure phase and proper functioning of the oxygen sensor, control unit and ventilation system was verified by *BioSpherix Ltd (Lacona*, *NY)* before further analysis.

### Brain and spinal cord pathology

Brains and spinal cords were harvested at P13 and P27 and immediately immersed in 4% paraformaldehyde for paraffin processing. Paraffin-embedded sections (5 μm) were stained with Luxol Fast Blue (LFB) and Periodic Acid—Schiff (PAS) to assess the general histology and myelination of these structures. Micrographs were prepared using an Olympus DP73 camera attached to an Olympus AX70 research microscope (Olympus America Inc., Center Valley, PA, USA) (n = 20 hypoxic animals P13; 14 normoxic animals P13; 4 normoxic animals P27; 4 hypoxic animals P27).

### Immunohistochemistry and Cell Counts

Brains harvested from mice were post-fixed overnight in 4% PFA, cryoprotected in 30% sucrose, processed for OCT embedding and sectioned at 40 μm as previously described [33]. For immunohistochemistry, standard methods with antigen retrieval in sodium citrate were used [34]. Antibodies used in this study included: anti-Olig2 (Millipore 1:500), anti-myelin basic protein (MBP; Millipore 1:500), anti-Cleaved Caspase 3 (CC3; Cell Signaling 1:500), and anti-Nkx2.2 (Iowa Hybridoma 1:50). Immunoreactivity was detected by incubation with appropriate Alexa-conjugated secondary antibodies (Molecular Probes—Life Technologies). Images of brain sections were captured using a Nikon NIE fluorescent microscope. The number of immune-positive cells per 100 μm^2^ area was quantified using Nikon Elements analysis software. Statistical significance was determined using Student’s t-test and presented as mean cells per field. All studies were blinded and performed on coded sections (n = 6 hypoxic + 6 normoxic animals for IHC and Western blotting (each)).

### Quantification of myelin-associated gene transcripts

Cerebral hemispheres of each animal were bisected, with one hemisphere used for measurement of gene-expression and the other for measurement of protein levels (described below). In analogy to nWMI as it is seen in human neonates (males display a more severe nWMI phenotype than females), molecular analyses beyond the weaning age of P21 were performed using the brains of male mice only.

For gene-expression studies, total RNA was extracted according to the manufacturer’s recommendations (TRIzol reagent, Life Technologies) and then was reverse-transcribed and amplified in one step (LightCycler 408, Roche Applied Science). Each reaction contained 12.5 μL of 2x master mix (QuantiFast SYBR Green RT-PCR Kit, Qiagen), 0.25 μL Quantifast RT Mix (Qiagen), 100 ng RNA, 1 μM of each forward and reverse primer (Table 1) in molecular grade water. Samples from each individual animal were run in duplicates, and the mean crossing point for each transcript was determined and normalized to *Gapdh* (deltaCt). DeltaCt values were used to calculate relative fold-change using the 2^-[delta][delta]Ct^ method [35]. Calculation of p-values used Student’s unpaired, two-tailed *t*-test (GraphPad 6, Prism); p < 0.05 was considered significant (n = 6 normoxic and 6 hypoxic animals P13; n = 6 normoxic and 6 hypoxic animals P27; n = 6 normoxic and 6 hypoxic animals P80, normoxic and hypoxic animals were from two independent experimental setups).

### Quantification of myelin-associated proteins

Total protein lysates was isolated from each cerebrum and brought to a concentration of 150 μg/μL in ice-cold lysis buffer (1x RIPA buffer supplemented with 10 mM NaF, 1 mM MgCl2, 100 μg/mL DNase I and a protease inhibitor cocktail (cOmplete, Roche)). Lysates were homogenized on ice by trituration through a 27-gauge needle before incubation for 30 minutes on ice. Detergent-insoluble material and brain lipids were removed by serial centrifugation (four rounds at 20,000 g for 10 minutes at 4°C).

For immuno-blotting, 150 μg brain tissue from each animal was loaded into each well of a 4–20% gradient gel and analyzed as previously described [36]. Thus, each lane represented an individual animal. Immunoblots were analyzed by densitometry (BioRad, Quantity One), with protein levels normalized to levels of β-actin. Student’s t-test or ANOVA compared normalized protein levels between experimental groups (Sigma Plot and Sigma Stat, Systat Software) (n = 8 normoxic and 8 hypoxic animals for western blot analysis at each time point: P13, P27 and P80). Hypoxic and normoxic animals were from two independent experimental setups per time point: 4 hypoxic + 4 normoxic animals from set 1, 4 hypoxic + 4 normoxic animals from set 2. All data analyses were performed in a blinded fashion (i.e., without knowledge of exposure and rearing assignment).

### Axon counts (thick sections)

6 month old animals were intracardially perfused with Trumps fixative. Spinal cords were dissected and cut into 1 mm thick blocks, oxidized with osmium, dehydrated and embedded in araldite. Blocks were cut into 2 μm thick sections, slide mounted and myelin stained with para-phenylene diamine (PPD). Microscopic images were taken at a 60x magnification from lateral and anterior funiculi of thoracic spinal cord sections and automated axon counts were performed as previously described [37]. As quality control, axon counts were verified manually from one slide per animal and compared to the software based outcome (animal numbers: 4 hypoxic and 4 normoxic animals).

### Myelin quality (g-ratios, dysmyelination)

Araldite embedded spinal cord blocks from 6 month old mice (see axon counts) were trimmed down to the lower anterior spinal cord funiculi (lower left or lower right quadrant) and sent to the Mayo electron microscopy core facility. Blocks were cut into thin sections, placed on carbon coated copper grids and stained with uranyl acetate. 20 representative electron microscopic images were taken per block (3000x or 8000x). The person taking the images was blinded to the experimental groups (JOW). Axon diameters, myelin thickness for g-ratios and dysmyelinated axons were determined from all axons per image using NIH *Image J* irrespective of the axon diameter with >100 axons per animal analyzed (n = 4 mice per group). In addition, we manually analyzed numbers of collapsed axons (whorls) and stressed axons (identified by intensively stained mitochondria) per treatment group.

### Neuro-motor assessment

All assessments of neurologic function were performed in a blinded fashion without knowledge of the rearing strategy of each experimental group. Male and female mice were tested separately. Similar to the human situation male mice had a more severe phenotype and were therefore the focus on all behavioral tests.

#### Hanging wire tests (single wire and mesh wire)

To evaluate motor function and limb strength, we performed two different hanging wire tests: for the mesh wire test, a mouse was placed in the center of a 50 x 50 cm wire grid, which was then gently inverted, and for the single wire test, an animal’s forepaws were placed at the center of a 2 m long single wire. Each attempt by the mouse to hang from the wire was considered a trial, which was completed either when the mouse fell, sustained its grip up to the cut-off point of 180 seconds or reached the end of the wire (in the case of the single wire) [38, 39]. Each mouse performed three trials per time point, with the best performances used for comparisons between exposure groups as previously described [38].

#### Rotarod test

To assess sensorimotor coordination, mice with no prior exposure to a rotating rod were placed upon a rod that was accelerated quickly from zero to five revolutions per minute, then gradually from five to 20 revolutions per minute. We considered each attempt by the mouse to remain on the rotating rod as a trial, completed when the mouse fell or had sustained itself on the rod up to the cut-off point of 180 seconds. We recorded the latency to fall for each of three trials and used the best performance for comparisons between exposure groups [40–43].

#### Grip-strength meter test

The effect of hypoxia on skeletal muscular strength was assessed by a grip-strength test [44]. The grip-strength apparatus (BioSeb, Chaville, France) consisted of a wire crossbar connected to an isometric force transducer or dynamometer. Male mice at P90 were lifted by their tails until their forepaws could grasp the grid. The mice were then gently pulled backward by the tail until the bar was released. The maximal force exerted by the mouse before losing grip was recorded. The mean of three measurements for each animal was calculated and normalized to the animal’s body weight, with the resulting data expressed as Newtons per gram (N/g) [45].

#### Spontaneous activity monitoring

Spontaneous locomotor activity was recorded with the Digiscan open field apparatus (Omnitech Electronics; Columbus, OH) and Versamax software, v.4.12-1AFE (Accuscan Instruments, Inc., Columbus, OH). The apparatus consists of six acrylic cages (40 × 40 × 30.5 cm) supported by a metal frame that holds two sets of photo cells. The device measures the number of discrete horizontal and vertical movements by tabulating the number of projected infrared beam interruptions. In all cages, mice were exposed to identical environmental conditions: freely accessible food and water, a normal 12 h light/dark cycle and 70°F ambient temperature. Groups of age-matched male CD1 mice (n = 3 for group responses, or n = 1 for individual response) were placed in the center of each cage at P90. Spontaneous activity was monitored over a period of six consecutive days, with data collected as number of beam breaks per 1 hour. The total horizontal and vertical activities were recorded using the Versadat software, v.3.02-1AFE (Accuscan Instruments) [46].

#### Body composition

Whole body composition (total fat mass and lean body mass) was determined by use of nuclear magnetic resonance imaging (MRI) technology produced by Echo Medical Systems LTD (Houston, TX). P90 male CD1 mice were placed in an MRI tube and analyzed on the accumulation 2 setting, which is specific for mice, as previously described [47].

### Statistical Analyses

The assumption of normality was tested with the Shapiro-Wilk test for normality prior to additional analysis (Sigma Plot v11.0). Normally distributed data were analyzed by Student’s unpaired, two-tailed t-test (2 groups) or ANOVA (> 2 groups). Data not normally distributed were analyzed using the Mann-Whitney U test (2 groups) or Kruskal-Wallis one-way ANOVA (> 2 groups). A probability of p < 0.05 was set as the level of significance for all comparisons.

## Results

### Four versus ten days of exposure to hypoxia improves survival and growth and does not require cross-fostering of neonatal mice

**Fig 1:**
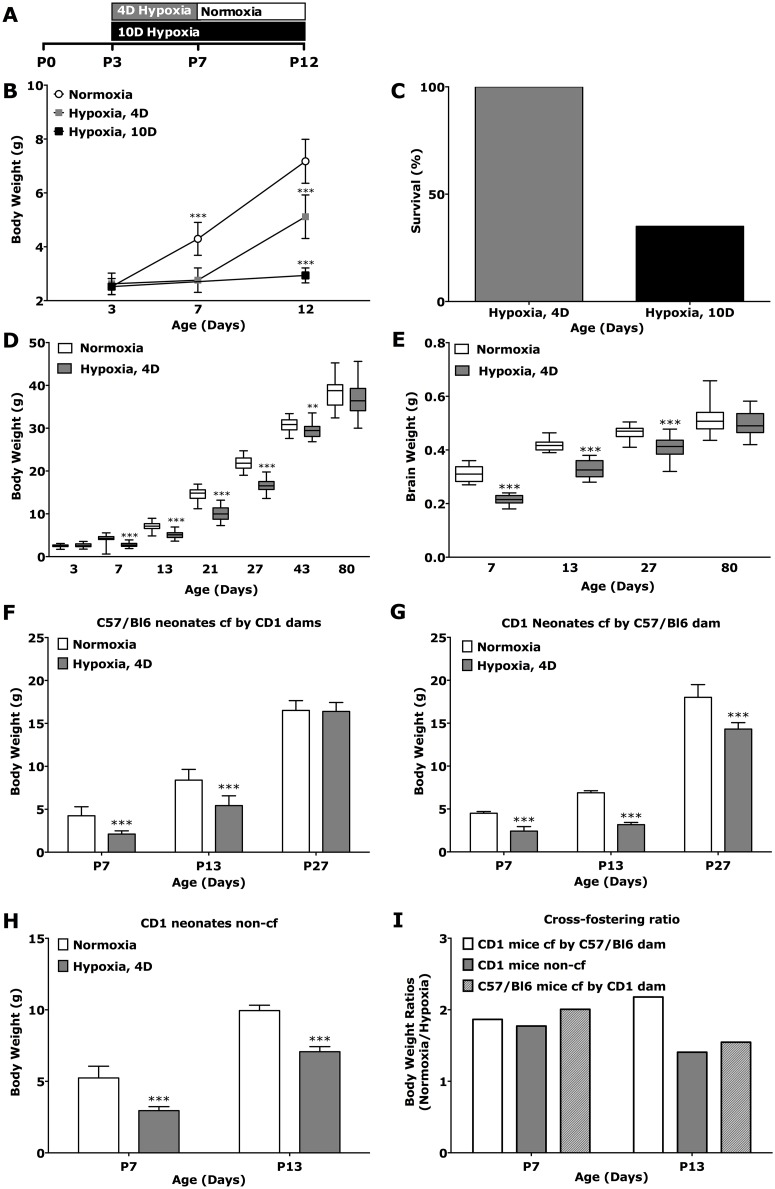
Comparison of growth and survival in models of neonatal hypoxic injury. A: Experimental timeline showing rearing strategies of neonatal CD1 mice under abbreviated hypoxia (red) (P3 → P7) followed by normoxia (gray), or long-duration hypoxia (green) (P3 **→** P12). B: Body weight comparison under abbreviated or long hypoxia (P3 **→** P7 or P3 **→** P12) (normoxia: n = 102; 10d hypoxia: n = 20; 4d hypoxia: n = 102). C: Survival rate of neonatal CD1 mice at P12 under abbreviated (red bar, n = 102) or long hypoxia (green bar), n = 20. D, E: Body (D) and (E) brain weight development of neonatal mice after hypoxia (P3 **→** P7) or normoxia (P3, n = 73 normoxic, 78 hypoxic; P7, n = 108 normoxic, 119 hypoxic; P13, n = 84 normoxic, 96 hypoxic; P27, n = 25 normoxic, 34 hypoxic; P43, n = 25 normoxic, 34 hypoxic; P80, n = 24 normoxic, 23 hypoxic mice). Litter sizes in A-E were 12 neonatal mice per dam. F-I: Body weight (F-H) and body weight ratios (I) in cross-fostered (F, G) and non-cross-fostered (H) normoxic and hypoxic (P3 **→** P7) CD1 and C57/Bl6 mice. Litter sizes in F were 6 neonatal mice per dam (non-cross-fostered CD1 mice: P7, n = 23 normoxic, 18 hypoxic; P13, n = 18 normoxic, 12 hypoxic mice; cross-fostered CD1 mice by C57/bl6 dam: n = 12 normoxic and 12 hypoxic mice for P7, P13 and P27; cross-fostered C57/Bl6 mice by CD1 dam: n = 12 normoxic and 12 hypoxic mice for P7, P13 and P27). Data are shown as mean ± std.-dev. *** p < 0.001; ** p < 0.01; * p < 0.05.

We first evaluated the effects of 4 days of neonatal hypoxia on growth and survival compared to 10 days of hypoxia using a litter size of 12 neonatal mice per dam. By postnatal day 12, 100% of the mice assigned to 4 days of hypoxia survived, while only 35% of the long hypoxia group survived (Fig 1, Table 1). Furthermore, mice in each experimental group displayed no gain in body weight during their exposure to hypoxia, but after their return to normoxia, mice in the 4 day hypoxia group had significant weight gain by P12 (Fig 1). Interestingly, the body weights and brain weights of mice immediately following hypoxia exposure at P7 (abbreviated duration mean = 2.76 g, Table 2) or at P12 (long duration mean = 2.90 g, Table 1) were not statistically different from P3 weights prior to treatment assignment (hypoxia mean = 2.63 g, normoxia mean = 2.52 g, Table 2). This data suggest a complete growth arrest in response to hypoxia. Given the apparent survival and growth benefit in abbreviated hypoxia, we followed subsequent litters of mice for extended periods of time to compare their body mass and brain growth compared to mice reared in room air. Hypoxic exposure impaired growth in body weight and brain weight for many weeks, although normalization did occur by adulthood (P80) (Fig 1; Table 2).

**Table 2:** Body and brain weights of mice exposed to abbreviated hypoxia.

	Body weight (g) (mean ± std.-dev.)		Brain weight (g) (mean ± std.-dev.)	
	10% O2	21% O2	10% O2	21% O2
**P3**—before assignment	2.63 ± 0.05	2.52 ± 0.04 (p = 0.071)		
**P 7**	2.76 ± 0.04	4.30 ± 0.06 (p < 0.001)	0.22 ± 0.005	0.31 ± 0.008 (p < 0.001)
**P13**	5.12 ± 0.08	7.18 ± 0.09 (p < 0.001)	0.33 ± 0.005	0.42 ± 0.004 (p < 0.001)
**P21 male**	9.27 ± 0.32	13.81 ± 0.35 (p < 0.001)		
**P27 male**	16.37 ± 0.35	21.71 ± 0.43 (p < 0.001)	0.41 ± 0.008	0.46 ± 0.005 (p < 0.001)
**P43 male**	29.52 ± 0.28	30.78 ± 0.32 (p = 0.004)		
**P80 male**	36.51 ± 0.92	38.12 ± 0.81 (p = 0.194)	0.50 ± 0.01	0.52 ± 0.01 (p = 0.338)

**Fig 2:**
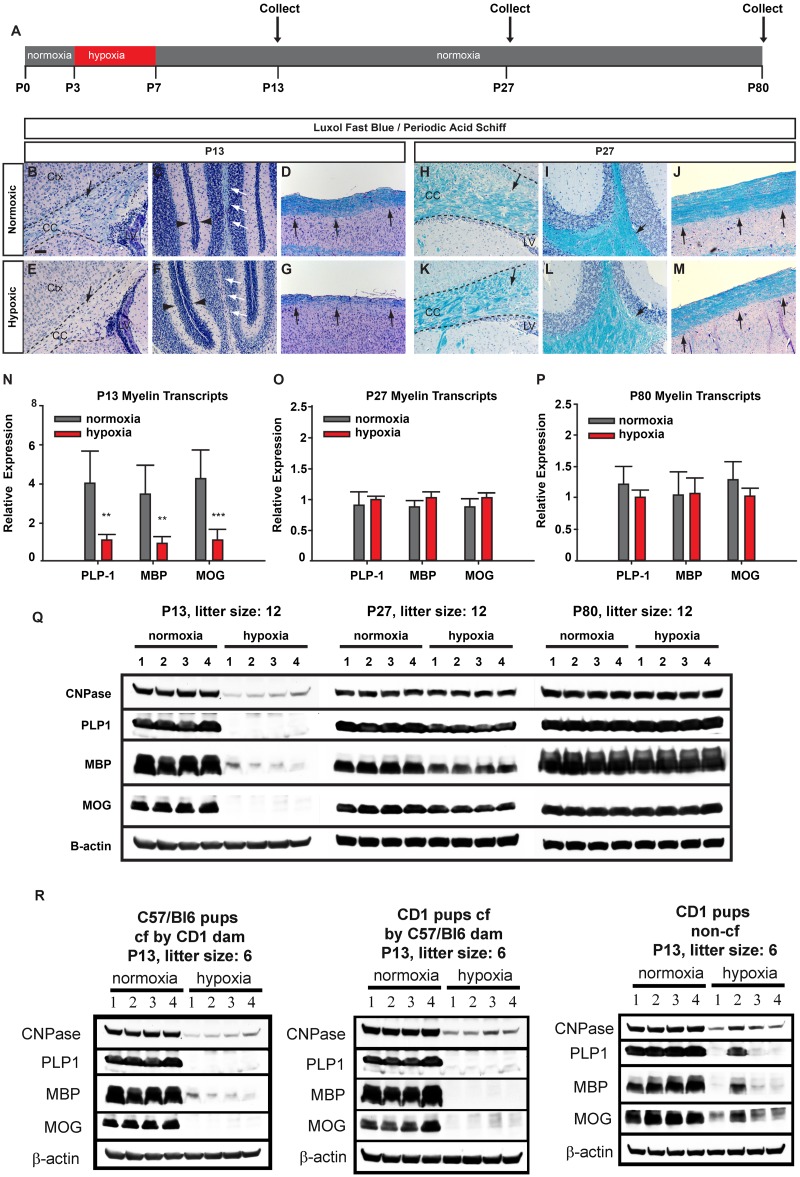
Abbreviated hypoxia is sufficient to induce CNS hypomyelination and does not require cross-fostering of neonatal mice. A: Experimental timeline showing CNS tissue collection from hypoxic (4 days of hypoxia) and normoxic CD1 mice at postnatal days 13, 27 and 80. B-M (Histology): Representative images of cerebrum (B, E, H, K), cerebellum (C, F, I, L) and spinal cord (D, G, J, M) of hypoxic and normoxic CD1 (non-cross-fostered) mice stained with myelin lipid dye Luxol fast blue (LFB) and Periodic acid—Schiff (PAS) (P13: B-G) (P27: H-M) at P13 and P27. At P13 black (cerebrum, spinal cord) and white (cerebellum) arrows indicate different levels of LFB staining in the corpus callosum (CC) respective differences in layer thickness in region matched areas from hypoxic and control mice. Black arrowheads indicate differences in external granular layer (EGL) thickness in cerebella between hypoxic and normoxic mice. At P27 black arrowheads indicate no obvious differences in LFB/PAS staining (H-M) between hypoxic and control CNS tissue in the corpus callosum, cerebellar white matter tracts and white matter in the spinal cord (n = 20 hypoxic animals P13; 14 normoxic animals P13; 4 normoxic animals P27; 4 hypoxic animals P27). N-P: RNA transcript levels of myelin splice variants PLP-1, MBP-1 and MOG from hypoxic or age-matched mouse control cerebra at P13, P27 and P80 shown as mean ± std.-dev. from 6 hypoxic + 6 normoxic animals per time point with *** equals p < 0.001; ** equals p < 0.01; * equals p < 0.05. Q: Representative Western blots showing protein levels of cerebral myelin markers CNPase, PLP, MBP and MOG (1.5 mg tissue loaded per lane) at P13, P27 and P80 with each lane representative for an individual animal. β-actin was used as a loading control. Litter sizes in A-Q were 12 neonatal mice per dam. R: Representative Western blots of myelin proteins identical to Q using total brain lysates from cross-fostered and non-cross-fostered CD1 and C57/Bl6 mice at P13. Litter sizes in R were 6 neonatal mice per dam. Ctx = cortex, CC = corpus callosum.

A known disadvantage of the more established hypoxia model is the need to co- or cross-foster hypoxic neonatal mice to avoid severe malnutrition or ultimately death. It was hypothesized that C57/Bl6 dams neglect their offspring when exposed to hypoxic stress [15, 23]. Although the survival rate in our model was 100% (therefore obviating the need for cross fostering in our paradigm), cross fostering could potentially impact post-hypoxic growth and therefore myelination. To eliminate the influence of litter size on nutrient availability we culled litters to 6 per group immediately prior to assignment to hypoxia at P3 (litter size for all other experiments mentioned in this manuscript is 12 with the exception of Fig 2R). To examine maternal impact on growth during and after 4 days of hypoxia, CD1 pups were raised with either a C57/Bl6 dam (cross fostered) or CD1 dam (non-cross fostered). In addition, we evaluated C57/Bl6 pups cross fostered with a CD1 dam. Experiments were also performed for all assignment groups under normoxia. We measured survival, body weight, and performed western blot analysis of myelin proteins (see following section) (Fig 1F–1I).

Surprisingly, the survival rates of neonatal mice in all assignment groups were identical (100% survival), including those litters fostered by C57Bl/6 dams. Body weight ratios of normoxic compared to hypoxic neonatal mice were similar in all assignment groups at P7 ((normoxic: hypoxic): 2.0 (cross-fostered C57/Bl6 pups), 1.9 (cross-fostered CD1 pups), 1.8 (non-cross-fostered CD pups)) but were significantly different at P13 ((normoxic: hypoxic: 1.6 (cross-fostered C57/Bl6 pups), 2.2 (cross-fostered CD1 pups), 1.4 (non-cross-fostered CD1 pups)). It is of note that non-cross-fostered CD1 mice showed the lowest body weight ratios of normoxic versus hypoxic mice at both time points, indicating the best growth rates post hypoxia (Fig 1F–1I).

It is of note that all CD1 dams lost approximately 25% of their body weight during the 4 day exposure to hypoxia compared to their body weight before hypoxia and compared to normoxic control dams (S1 Fig). This data suggested that the dams are likely responsible for malnourishment of neonatal pups under hypoxia and strongly questions co- or cross-fostering of neonatal mice during the hypoxic treatment.

In brief, results demonstrate improved survival, body, and brain growth under four days of hypoxia compared with 10 days of hypoxia. Body and brain weights of neonatal mice were lower in the hypoxic groups until mouse adulthood. No obvious beneficial effect of cross-fostering on survival rates or weight loss was observed during the 4 day hypoxic insult without obvious differences between mouse strains. However, weight gain of neonatal mice post injury occurred faster with CD1 dams. Importantly, non-cross-fostered neonatal CD1 mice demonstrated the best weight gain post hypoxia with the smallest interference of malnutrition.

### Four days of hypoxia reduces myelination and the expression of myelin transcripts

To determine whether 4 days of hypoxic exposure was sufficient to induce neuropathological changes similar to nWMI, we assessed the cortex and spinal cord morphology. Cortical thickness was decreased in hypoxic animals (S2 Fig). LFB staining of myelin-specific lipids was reduced in the corpus callosum, cerebellum and spinal cord at one week post hypoxic insult (P13) (Fig 2B–2G). Myelination had improved by P27, though subtle differences in the extent or intensity of staining persisted (Fig 2H–2M). In addition, the cerebellar external granular cell layer (EGL) thickness at P13 was increased in hypoxic animals suggesting delayed cerebellar development (Fig 2C and 2F).

To quantify the extent to which hypoxia reduced the elaboration of cerebral myelin, we measured the expression levels of myelin-associated gene transcripts and their protein products. Hypoxia strongly down-regulated the gene expression of myelin markers proteolipid protein 1 (PLP1), myelin basic protein (MBP) and myelin oligodendrocyte glycoprotein (MOG) at P13 (Fig 2N, Table 3, S1 Table). Interestingly, the expression of these myelin-associated genes did normalize at later time points (Fig 2O and 2P, Table 3, S1 Table). As expected, protein levels for these three myelin markers were markedly reduced at P13, as was the level of the immature OL marker 2',3'-cyclic-nucleotide 3'-phosphodiesterase (CNPase) protein (Fig 2Q, S2 Table). However, the levels of these proteins remained depressed through P27 and did not normalize until approximately P80 (Fig 2Q, S3–S4 Tables). We also analyzed the acute effects of hypoxia on the OL lineage at P7 directly after the hypoxic injury. The immature OL marker CNPase was down-regulated already at P7 directly after the hypoxic insult. OL markers that indicate later stages of OL lineage progression (PLP, MBP, MOG) were undetectable at P7 (S5 Table).

**Table 3:** Fold-change in gene transcripts at P13, as detected by RT-PCR.

gene	Fold change under hypoxia P13	Fold change under hypoxia P27	Fold change under hypoxia P80
PLP-1	0.26 (p value: 0.001)	1.13 (p value: 0.171)	0.84 (p value: 0.159)
MBP	0.27 (p value: 0.003)	1.18 (p value: 0.860)	0.98 (p value: 1.00)
MOG	0.26 (p value: 0.001)	1.18 (p value: 0.269)	0.87 (p value: 0.385)

Identical to rearing assignments described above (Fig 1F) with cross-fostered C57/Bl6, cross-fostered CD1 mice and non-cross-fostered CD1 mice, we further analyzed the impact of malnutrition on hypomyelination by correlating brain/body weights (Fig 1F) with levels of myelin proteins in hypoxic mice (litter size was 6 for all assignments) (Fig 2R). CNS myelination at P13 was determined by Western blotting and strongly correlated with brain and body weights from hypoxic and normoxic mice (Fig 1F). Cross-fostered CD1 mice showed the highest ratios of myelin proteins between normoxic and hypoxic animals (normoxic: hypoxic, CNPase: 10.0; MBP: 136.2; PLP: 293.7) followed by cross-fostered C57/Bl6 mice (normoxic: hypoxic, CNPase: 8.2; MBP: 39.1; PLP: 9.1) and non-cross-fostered CD1 mice (normoxic: hypoxic, CNPase: 1.1; MBP: 1.7; PLP: 1.5) (Fig 2R).

Of significance, brain/body weight ratios between normoxic and hypoxic animals in different rearing assignments strongly correlated with myelin protein ratios at P13 and suggest an underlying effect of malnutrition on hypomyelination 1–2 weeks post injury.

### Abbreviated exposure to hypoxia causes reduced OPC levels and substantial apoptosis

**Fig 3:**
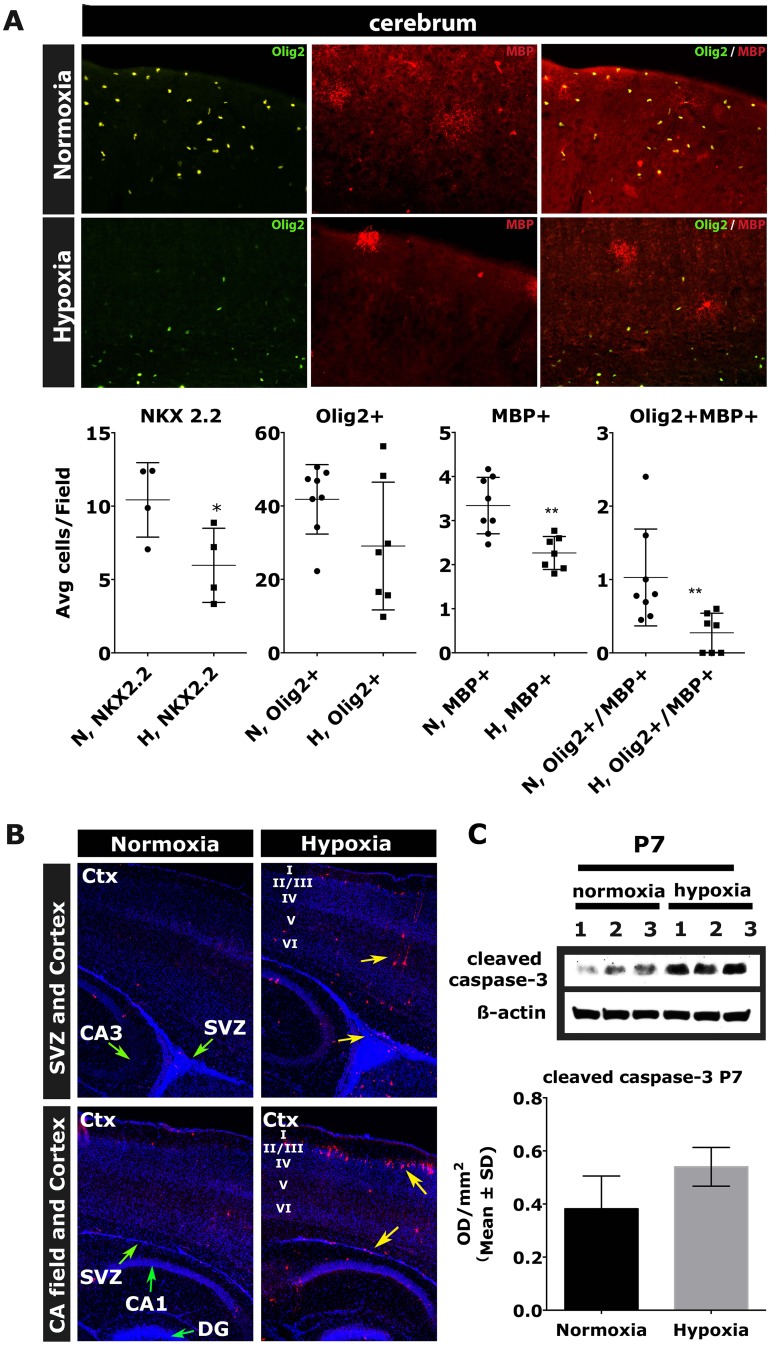
Abbreviated hypoxia (P3 → P7) does not increase levels of OPCs but causes substantial apoptosis throughout cortical layers, hippocampus and SVZ. A: Immunohistochemistry and stereologic analysis of mouse cerebra at P13 using OL markers Olig-2 (OPCs, immature OLs, mature OLs), MBP (mature OLs) and NKX2.2 (OPCs). B: Immunohistochemical staining of level matched hypoxic and control cerebra at P7 showing anti-CC3 (red) and nuclear marker DAPI (green arrows indicate specific regions SVZ, DG and CA1/3 field; yellow arrows mark levels of high apoptotic intensity in hypoxic mice). C: Representative Western blots using total brain homogenates from hypoxic and control CD1 mice at P7 using apoptosis marker CC3 and β-actin as a loading control. Densitometric analysis of Western blots from 3 independent experiments showing brain levels of CC3 at P7 in hypoxic and control mice with *** equals p < 0.001; ** equals p < 0.01; * equals p < 0.05.. SVZ, subventricular zone; DG, dentate gyrus; CA1-3, hippocampal CA fields; Ctx, cortex, I-VI = cortical layers 1–6. (n = 6 hypoxic + 6 normoxic animals for immunohistochemistry and Western blotting (each)).

An important question was whether CNS hypomyelination post hypoxia is caused by oligodendrocyte maturation arrest with increased levels of OPCs, or whether death of CNS cells including OL lineage cells is responsible for the effect seen. We first performed cell counts of Olig2 (+) and MBP (+) cells of the OL-lineage at P13 in the rostral and caudal cerebrum including the extent of double-labeled Olig-2 (+)/MBP (+) cells. Olig-2 is a marker for late OPCs, immature and mature OLs, while MBP specifically labels mature OLs only. MBP (+)/Olig2 (+) double-labeled cells are therefore mature OLs. Whole brain quantitation of cell counts revealed reduced numbers of Olig-2 (+) and MBP (+) cells in the hypoxic group. Differences were significant for mature MBP (+) cells. Similarly, levels of MBP (+)/Olig2 (+) double-labeled cells (mature OLs) were significantly reduced in the hypoxic group throughout the entire cerebrum (Fig 3A). Results indicated lower levels of mature MBP (+) OLs as suggested by Western blot analysis from whole brain at the same time point. It was unclear, however, whether reduced numbers of Olig-2 (+) cells under hypoxia were based on mature OLs only or due to reductions in numbers of OPCs and immature OLs as well. We therefore performed cerebral cell counts using the OPC marker NKX2.2 showing significantly reduced levels of OPCs in the hypoxic group at P13 (Fig 3A).

To further address this question we performed Western blots from the entire brain using four different OPC markers Olig-1, Olig-2, PDGFαR and NG2 at P7, P13, P27 and P80. The late OPC marker NG2 and the OPC/immature/mature OL marker Olig-2 were significantly reduced at P7 (S3 Fig, S5 Table). Slight, albeit not significant, reductions in levels of OPC markers Olig-1 and PDGFRα at P7 were also noted. At postnatal day 13, OPC markers PDGFRα, Olig-1 and Olig-2 were reduced in cerebral tissue from hypoxic mice, but their levels all had normalized by P27 (S3 Fig, S2 and S3 Tables). Levels of NG2 protein were similar in the exposure groups at P13 and P27 and were actually higher in the hypoxia group at P80 as were levels of Olig-1 at P80 (S3 Fig, S2, S3, S4 Tables).

In summary, cell counts and Western blot analysis showed no increase in numbers of OPCs/OPC markers, which might be expected in case of an OPC differentiation block. Instead, both sets of data indicated moderate reductions of OPCs/OPC markers.

We next assessed levels of apoptosis in the forebrain using the marker cleaved caspase-3 (CC3). Western blot analysis using whole brain lysates at P7 revealed increased levels of CC3 in the hypoxic group relative to control animals (Fig 3C). To further characterize which brain regions were most impacted by the hypoxic insult we performed immunofluorescent staining with CC3. Morphological analysis directly after hypoxia at P7 revealed an increase in CC3 (+) cells in many brain regions, most notably in cortical layers 2 and 5, the hippocampus and SVZ progenitor compartment. Differences in CC3 were not detected by Western blot analysis at later time points (P13 and P27) (Fig 3B, S5 Table).

These results demonstrate that abbreviated hypoxia affects levels of OPCs (Fig 3A, S3 Fig), immature OLs (Fig 2Q and 2R) and mature OLs (Fig 2Q and 2R, Fig 3A). Reductions in OPC numbers and OPC markers for longer 6 days post-injury do not support the hypothesis of an oligodendrocyte differentiation arrest in this model. Instead, we demonstrate substantial cell death post-injury throughout the entire brain and particularly present in cortical projection neurons of layers 2 and 5 and in the SVZ.

### Abbreviated exposure to hypoxia impairs motor function and activity

**Fig 4:**
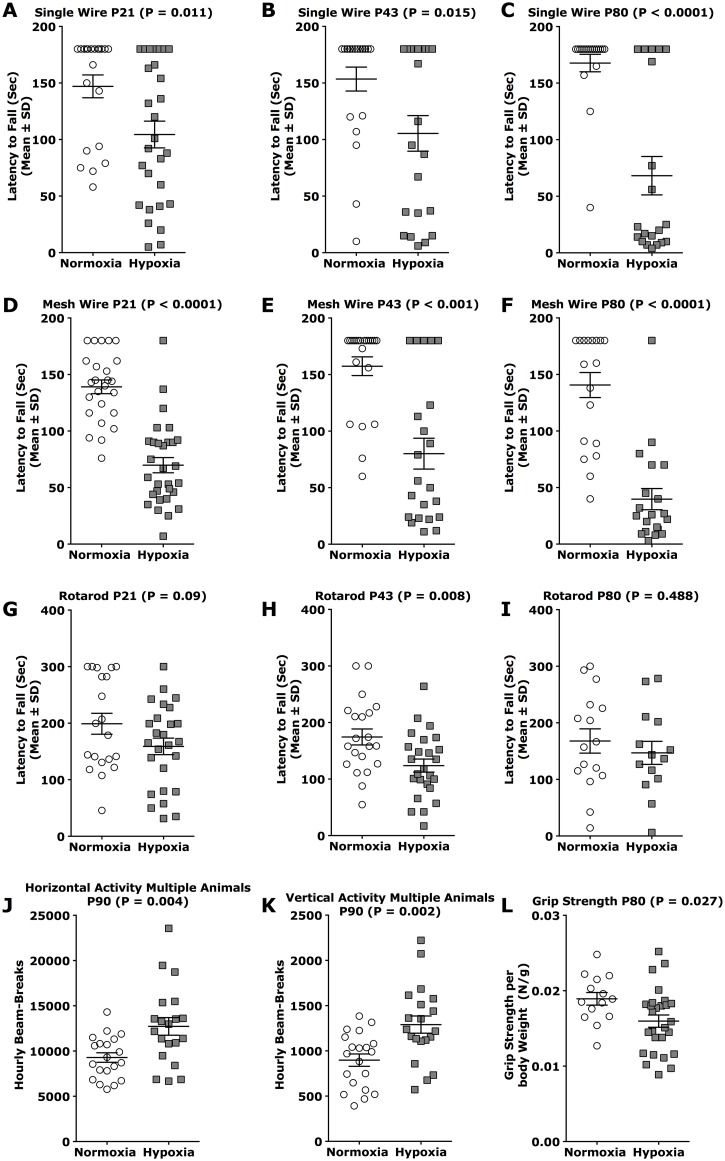
Abbreviated hypoxia is sufficient to induce persistent motor-deficits in mice. Mice reared under hypoxia (P3 **→** P7) or room air were tested at weaning age (P21), early adulthood and adulthood (P43, P80) for motor coordination and strength (D-F), strength (A-C), balance and endurance (G-I), front limb grip strength (L), body composition (E, F), global nocturnal activity in groups of three mice per box (J, K) and global nocturnal activity of single mice per box (I, J). A-C: hanging wire-single test at P21 (A), P43 (B), P80 (C); D-F: hanging wire-mesh test at P21 (D), P43 (E), P80 (F); G-I: Rotarod test at P21 (G), P43 (H), P80 (I). J, K: Global nocturnal activity of grouped mice (3 per activity box) showing horizontal hourly beambreaks (J) and vertical hourly beambreaks (K) for 5 nights from P91-P96. L: Grip strength meter test at P90. N per test and time-point >34 animals for hanging wire tests, Rotarod and grouped nocturnal activity; N = 14 per group for grip strength meter test with *** equals p < 0.001; ** equals p < 0.01; * equals p < 0.05.

Next we assessed motor coordination and strength in the hypoxia cohort of male mice (non-cross-fostered CD1 mice only). Our focus was on male mice because the motor performance of male mice worsened during adulthood (P43 until P80), which has been described by other investigators [16]. In contrast, female mice improved during adulthood (P43 until P80) or remained on similar performance levels (Fig 4, Table 4, S6 Table).

**Table 4:** Behavioral motor testing—Male mice.

	Latency to fall (s) (mean + std.-dev.)	
Motor test	Normoxia, M	Hypoxia, M
**P21, mesh wire**	139.1 ± 30.3, (n = 25)	69.8 ± 36.9, (n = 30) (p < 0.001)
**P21, single wire**	147.0 ± 46.3, (n = 21)	104.4 ± 62.4, (n = 28) (p = 0.011)
**P21, Rotarod**	199.0 ± 82.7, (n = 20)	158.9 ± 74.6, (n = 26) (p = 0.092)
**P43, mesh wire**	157.4 ± 38.8, (n = 22)	80.1 ± 64.0, (n = 22) (p < 0.001)
**P43, single wire**	153.5 ± 49.7, (n = 22)	105.4 ± 73.8, (n = 22) (p < 0.016)
**P43, Rotarod**	174.5 ± 64.7, (n = 21)	123.8 ± 57.8, (n = 25) (P = 0.008)
**P80, mesh wire**	140.7 ± 49.5, (n = 20)	39.8 ± 42.0, (n = 20) P < 0.001
**P80, single wire**	167.7 ± 33.8, (n = 19)	68.2 ± 75.9, (n = 20) (p < 0.001)
**P80, Rotarod**	179.1 ± 44.5, (n = 17)	158.1 ± 78.4, (n = 25) (p = 0.200)
**Grip strength meter test P90**	0.019 ± 0.003 (N/g), (n = 14)	0.016 ± 0.004 (N/g), (n = 27) (p = 0.027)
**Global nocturnal activity—horizontal (groups of 3 mice per box)**	9283 ± 2355 hourly beam breaks (n = 20)	12726 ± 4360 hourly beam breaks (n = 20), (p = 0.004)
**Global nocturnal activity—vertical (groups of 3 mice per box)**	898 ± 303 hourly beam breaks (n = 20)	1291 ± 425 hourly beam breaks (n = 20), (p = 0.002)
**Global nocturnal activity—horizontal (1 mouse per box)**	3708 ± 865 hourly beam breaks (n = 6)	6026 ± 1420 hourly beam breaks (n = 6), (p < 0.001)
**Global nocturnal activity—vertical (1 mouse per box)**	342 ± 163 hourly beam breaks (n = 6)	642 ± 292 hourly beam breaks (n = 6), (p < 0.001)

In hanging wire tests assessing coordination, strength and endurance male mice exposed to hypoxia displayed shorter grip latencies at P21, P43 and P80 (Fig 4A–4C **single wire**; Fig 4D–4F **mesh wire**; Table 4). Similarly, hypoxia significantly reduced the latency to fall from the rotarod at P43 (Fig 4H; Table 4). In contrast to the behavioral outcome from both hanging wire tests at P80, no differences were detected between hypoxic and control animals in rotarod performance at this time point. To assess forelimb grip strength we used the grip strength meter test. Grip strength was reduced by 16% in adult hypoxic mice at P90 (Fig 4L; Table 4). Body weight and MRI-measured lean muscle mass were similar in the two experimental groups (S4C and S4D Fig; Table 4); indicating motor performance was not secondary to differences in body composition. Beginning at P90, we monitored the global nocturnal activity of mice from each experimental group for six days. Hypoxic mice displayed increased horizontal and vertical activity, as evidenced by more frequent horizontal (normoxia mean = 9283; hypoxia mean = 12,726) and vertical (normoxia mean = 898; hypoxia mean = 1,291) beam-breaks (Fig 4J and 4K; Table 3). To determine whether social interactions or intrinsic hyperactivity of hypoxic mice was responsible for this effect, testing was repeated with individual mice under otherwise identical conditions. Interestingly, the results of these experiments were even more pronounced than those described above (S4A and S4B Fig; Table 3). Thus, along with motor and strength deficits, mice exposed to hypoxia displayed signs of abnormal hyperactivity as well.

### Abbreviated exposure to hypoxia causes dysmyelination of spinal neurons and changes axonal composition in spinal cords

**Fig 5:**
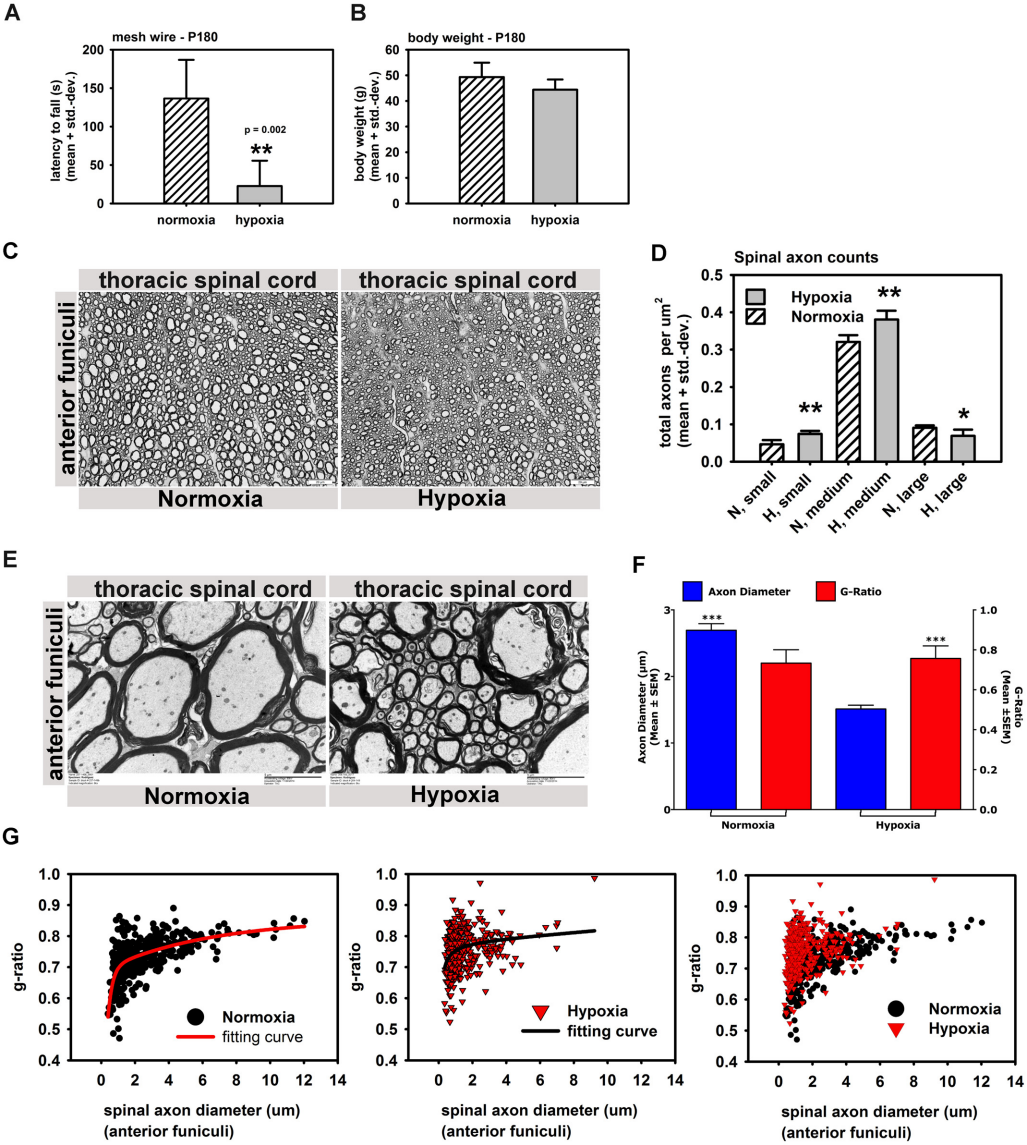
Abbreviated hypoxia changes axonal composition in spinal cords and causes dysmyelination of spinal axons. A, B: 6 month old hypoxic mice showed strong motor deficits in hanging wire tests (A) without having different body weights (B) (n = 4 normoxic + 6 hypoxic animals). C: Spinal cord thick sections from anterior and lateral funiculi (thoracic regions) in hypoxic and normoxic mice 6 month after the hypoxic insult (4 control + 4 hypoxic mice). D: Automated axon counts of spinal cord thick sections from C. showing abnormal axonal compositions in spinal anterior funiculi with increased small and medium diameter axons and decreased numbers of large diameter axons relative to normoxic controls (small = 1–4 μm; medium = 4–10 μm; large = > 10 μm) (4 control + 4 hypoxic mice). E: Electron microscopy of spinal cord sections left and right of the anterior median fissure (thoracic spinal cord) in hypoxic and control animals (magnification: 8kx). Higher g-ratios (thinner myelin sheaths) and loosely wrapped myelin around axons was prominent by Electron microscopy in thoracic spinal motor neurons (anterior funiculi) (4 control + 4 hypoxic mice, 100 axons per animal). F. Chi-Square analysis of the g-ratio/axon diameter relationship in hypoxic and normoxic animals. G: Scatter plots of g-ratio vs. axon diameter in control (black) and hypoxic animals (red) with best fitting curves, with *** equals p < 0.001; ** equals p < 0.01; * equals p < 0.05.

CNS hypomyelination post hypoxia is a potential cause for neuro-motor deficits in nWMI models. However, while myelin levels catch up biochemically within weeks post injury motor deficits persist in animals. Cerebral dysmyelination with thinner myelin sheaths or increased g-ratios (the ratio of axon circumference to myelin circumference) and imperfect myelin wrapping around axons was recently discovered in adult mice reared under hypoxia and served as an explanation for persistent neuro-motor deficits [14]. To confirm this finding and further characterize the impact of hypoxia on spinal cord dysmyelination, we assessed "myelin quality" (g-ratios, axon diameters and proper myelin wrapping) in 6 month old hypoxic and normoxic CD1 mice. We first confirmed the severe motor phenotype in all hypoxic mice using hanging wire tests (Fig 5A) with no significant differences in body weights between both groups (Fig 5B). Thick sections of thoracic spinal cords from all animals were analyzed for overall spinal cord preservation of perfused animals without obvious squeeze artifacts. Surprisingly, the spinal axon composition in anterior funiculi containing motor and efferent pathways including the vestibulospinal tract (stimulates axial extensor muscles), the anterior corticospinal tract (control of voluntary, skilled movements), and the tectospinal tract (mediates reflex movements in response to visual stimuli) was visibly different between both assignment groups [48–51].

Hypoxic animals showed lower numbers of large diameter axons (small diameter: 1–4 μM; medium diameter: 4–10 μM, large diameter: > 10 μM) in anterior and lateral funiculi of the thoracic spinal cord (Fig 5C). Automated (software-based) quantitation (60x images) from spinal anterior and lateral funiculi (throracic region) confirmed our qualitative assessment and demonstrated not only significantly fewer large diameter axons in hypoxic mice but at the same time more small and medium diameter axons compared to normoxic control mice born on the same day (Fig 5D). Electron microscopy of 800 spinal axons (400 hypoxic, 400 normoxic, 100 axons per animal) left and right of the anterior median fissure from adult hypoxic and normoxic animals and subsequent data analysis (Sigma Plot) indicated a) absence of the Normality criterion for the data distribution (*Shapiro-Wilk Test*) and b) highly significant differences in the g-ratios as well as axon diameters between the hypoxic and normoxic group with p< 0.001 (each) using the *Mann-Whitney Rank Sum Test* and the *Kruskal-Wallis One Way Analysis of Variance on Ranks*. A Contingency test (Chi-square, two-sided) analyzing the data distribution of g-ratio versus axon diameter in hypoxic and normoxic animals resulted in a highly significant difference between both datasets (p < 0.0001, Odds ratio: 1.840) (Fig 5E and 5F). The axon diameters (median) were 2.12 μm in the normoxic group and 1.13 μm in the hypoxic group while g-ratios (median) were 0.742 for normoxic animals and 0.759 for hypoxic animals (Fig 5F). Smaller g-ratios found in control animals are equivalent with thicker myelin sheaths compared to those in hypoxic mice. No significant difference was found in the number of whorls (collapsed, apoptotic axons), or axons with intensively stained mitochondria between both groups. The data shown in Fig 5G suggested a non-linear relationship between g-ratio and axon diameter in small diameter axons and a rather linear relationship in large diameter axons. In an attempt to identify the best fit for the data distribution in both groups we used a non-linear equation (*Sigma Plot)* for the normoxic group: "Exponential Rise to Maximum, Double, 5 Parameter" with f = y0+a*(1-exp(-b*x))+c*(1-exp(-d*x)); R = 0.614, R^2^ = 0.377; Standard Error of Estimate: 0.053. Logarithmic functions (3rd function) and Ligand binding functions (one and two site saturation) had a similar outcome with slightly reduced R^2^ values. Importantly, using the same functions for the data distribution in the hypoxic group resulted in a poor fit with R^2^ values of 0.1 or below (Fig 5G), which indicated a different relationship between g-ratio and axon diameter in the spinal cord of hypoxic animals.

In summary, results indicate that hypoxia from P3 until P7 is sufficient to cause long-term myelination deficits in 6 month old mice. In addition, we demonstrate spinal white matter changes affecting tracts responsible for motor function. Both myelination deficits and axonal composition in the spinal cord strongly correlate with the persistent motor phenotype in adult animals.

## Discussion

nWMI underlies the neurodevelopmental delays often seen in children born at the extremes of prematurity with diffuse hypomyelination, reduced cortical white matter and increased ventricle sizes as characteristic disease markers [4].

In nWMI patients, it is suspected that hypoxia and recurrent episodes of mild hypoxia-ischemia impair or delay OPC differentiation or alternatively induce OPC cell death [7, 8], which in turn results in dysmyelinated and likely dysfunctional axons. Recent studies in transgenic animals modeled the underlying neuropathology of nWMI through hypoxia and rescued the severe neuro-motor phenotype through stimulation of OPC proliferation, thereby resulting in lower levels of dysmyelinated axons [14, 25]. Together these studies highlight the importance of OL-lineage cells and their interaction with neuronal cell types for disease pathology and future interventional strategies.

A persistent neurological phenotype has been described in C57BL/6 mice exposed to the longer, standard period of hypoxia [10, 14]. However, it was previously unclear whether and, to what extent, outbred CD1 mice are susceptible to hypoxic stress [26]. Our data demonstrate that CD1 mice suffer from significant motor and behavioral abnormalities that persist well into adulthood after only 4 days of hypoxia during the neonatal period. Recent electron microscopic studies have demonstrated a quantitative difference of myelin in the cerebral cortex during adulthood [14, 25] with higher g-ratios of myelinated axons (thinner myelin sheaths) and imperfectly wrapped myelin around axons. We confirm higher g-ratios in spinal white matter tracts responsible for motor function. Loosely wrapped myelin around axons was found in the white matter of both hypoxic and control spinal cords (possible fixation artifact), however, to a higher extent in hypoxic animals.

Mechanistically, it is undetermined whether the OPC/OL differentiation arrest and/or cell death of OPCs are responsible for CNS dysmyelination and motor deficits seen in our model of nWMI and patients with the disease. Our data shown here does not favor the OPC lineage arrest hypothesis, which would show an accumulation of OPCs that are unable to differentiate into immature and mature OLs. Instead, we demonstrate moderately reduced OPC numbers and reduced levels of OPC markers at different time points post hypoxia throughout the entire brain. As an alternative explanation, animals reared under hypoxia revealed substantially increased levels of apoptosis post injury at P7 in CA fields, SVZ and cerebral cortex relative to controls. Specific location and morphology of apoptotic cells indicates an involvement of cortico-spinal projection neurons. Indeed, histological analysis of the adult spinal cord demonstrates substantial changes in the spinal neuron composition in animals reared under hypoxia. Changes were particularly pronounced in white matter tracts in anterior funiculi associated with motor outcome including the vestibulospinal tract, the anterior corticospinal tract and the tectospinal tract [48–51]. We hypothesize that axonal changes together with myelination deficits are responsible for the persistent motor phenotype observed in adult CD1 mice. In support of this hypothesis we demonstrate a strong correlation between motor phenotype and abnormal spinal composition plus dysmyelination. All animals with abnormal spinal composition plus dysmyelination revealed substantial motor deficits in hanging wire tests and vice versa. However, the ability and the extent of affected neurons to function normally has yet to be determined.

The underlying neuropathology of nWMI is at least partially reproduced in animal models applying long-duration hypoxia to neonatal mice for up to 11 days beginning at P3 [10, 14, 15, 18, 23, 24, 26]. However, this rodent model may inaccurately recapitulate the human disease from a neurodevelopmental perspective; the OL population shifts from its majority of late OPCs (A2B5+, O4+) at the outset to a preponderance of immature OLs (O4+, O1+, CNPase+) by the end of the exposure period [52–54]. Furthermore, such prolonged hypoxia impairs growth and survival in neonatal mice, confounding the ability to accurately interpret these models. Existing mouse models require co- or cross-fostering to avoid death and severe malnutrition of neonatal mice, thereby adding another level of complexity and additional costs. Inbred C57/Bl6 mice are typically used as a model strain due to their proposed higher sensitivity to hypoxic stress [26] irrespective of their low genetic variability with limited prognostic value for the human disease.

In the present study, we demonstrate that exposure to hypoxia from P3 until P7, which corresponds to the developmental phase of the human brain between gestational weeks 32 and 36, is sufficient to induce CNS hypomyelination for 1–2 weeks post hypoxia and persisting motor and neurobehavioral disturbances combined with spinal dysmyelination and changes in the axonal spinal composition in adult outbred CD1 mice, without compromising survival. While we cannot directly equate our findings with similar hypoxia models due to differences in exposure time and strain [14, 15, 23, 25], it is notable that the reduction in myelin protein expression and delay in myelination through P27 is comparable to or exceeds what has been reported previously [14, 15, 23, 25].

We also demonstrate that cross-fostering of neonatal CD1 or C57/Bl6 mice has no obvious impact on weights or survival during the assignment to hypoxia in our shortened model of nWMI with hypoxia inducing an complete growth arrest in neonatal mice. Non-cross-fostered CD1 mice showed the lowest level of growth retardation post hypoxia compared to both cross-fostered CD1 and cross-fostered C57/Bl6 mice. In our hands, the level of growth impairment directly correlated with the level of hypomyelination observed among the rearing assignments. It is unclear whether hypomyelination is a mere by-product of malnutrition or the combined outcome of hypoxia and malnutrition. It is well known that early postnatal starvation of rodents in the absence of hypoxia causes severe CNS hypomyelination [55], delayed oligodendrocyte differentiation [56] and disturbed glial proliferation [57, 58]. Based on this data we speculate that hypomyelination 1–2 weeks post hypoxia (P7 until P27) is more likely induced by malnutrition rather than a direct outcome of low oxygen levels. To minimize the impact of malnutrition in this model we substantially reduced the exposure time to hypoxia from 10 days to 4 days and used non-cross-fostered CD1 mice, which showed the best rates of body and brain weight gain post hypoxia compared to cross-fostered C57/Bl6 mice and cross-fostered CD1 mice.

In summary, we provide detailed characterization of a novel model of nWMI in which an abbreviated course of hypoxia targeting the neonatal brain of outbred CD1 mice induces hypomyelination, a persistent motor phenotype, adult dysmyelination and permanent changes in the spinal axon composition. The latter finding may be a useful future pathological marker also for the human situation, and may aid in development of therapeutics for nWMI. The added benefits of our nWMI model include a more specific exposure time to hypoxia that corresponds well with human development, decreases murine neonatal death, diminishes weight loss/malnourishment, and eliminates cross-fostering. Importantly, the use of CD1 mice affords large litter sizes (relative to C57BL/6 mice) and reduces research costs due to lack of co- or cross-fostering, which enables large-scale, preclinical screening, development, and testing of therapeutic compounds. This abbreviated hypoxia animal model may be appropriate for future *in vivo* studies of nWMI, especially those focusing on therapeutic interventions.

## Supporting Information

S1 Fig Abbreviated hypoxia causes substantial body weight loss in CD1 dams. Body weights of timed pregnant CD1 dams (Charles River Laboratories) were determined right before the assignment to hypoxia or normoxia (when neonatal pups were 3 days old) and after the assignment to hypoxia or normoxia (when neonatal pups were 7 days old) (n = 12 per group). Results indicate a 25% body weight reduction in dams assigned to 4 days of hypoxia (10% O2) compared to dams in room air (normoxia) with *** equals p < 0.001; ** equals p < 0.01; * equals p < 0.05.
(TIF)

S2 Fig Abbreviated hypoxia (P3 → P7) reduces cortical thickness. Representative cortical images from hypoxic and normoxic brains (P7) illustrating measurement of cortical thickness in level matched tissue (yellow line indicates the cortical thickness measured). Cortical thickness was determined at multiple locations per brain and quantified as mean ± std.-dev. with *** equals p < 0.001; ** equals p < 0.01; * equals p < 0.05.
(TIF)

S3 Fig Densitometric Western blot analysis of OPC markers. Densitometric analysis of Western blots from 3 independent experiments using total brain lysates from hypoxic and control CD1 mice at P7, P13, P27 and P80. Bar graphs show levels of OPC markers PDGFαR, NG2, Olig-2 and Olig-1.
(TIF)

S4 Fig Additional behavioral and body mass analysis. Mice reared under hypoxia (P3 **→** P7) or room air were tested during adulthood for global nocturnal activity (**A, B**) and body mass analysis (**C, D**). **A, B**: Global nocturnal activity with single mice per box (n = 6 hypoxic + 6 normoxic mice) showing horizontal hourly beambreaks **(A)** and vertical hourly beambreaks (**B**) for 5 nights from P91-P96. C, D: Echo-MRI analysis of lean body mass (**C**) and fat content (**D**) in hypoxic and control mice, *** equals p < 0.001; ** equals p < 0.01; * equals p < 0.05.
(TIF)

S1 Table RT-PCR primers. RT-PCR primers used to determine mouse CNS mRNA levels at P13, P27 and P80.
(DOCX)

S2 Table Densitometric analysis of Western blots from cerebrum at P13. Densitometric analysis of Western blots from mouse cerebra at P13 (n = 8 hypoxic and 8 normoxic mice). Calculation of p-values used Student’s unpaired, two-tailed *t*-test (Sigma Plot 11.0); p < 0.05 was considered significant.
(DOCX)

S3 Table Densitometric analysis of Western blots from cerebrum at P27. Densitometric analysis of Western blots from mouse cerebra at P27 (n = 8 hypoxic and 8 normoxic mice). Calculation of p-values used Student’s unpaired, two-tailed *t*-test (Sigma Plot 11.0); p < 0.05 was considered significant.
(DOCX)

S4 Table Densitometric analysis of Western blots from cerebrum at P80. Densitometric analysis of Western blots from mouse cerebra at P80 (n = 8 hypoxic and 8 normoxic mice). Calculation of p-values used Student’s unpaired, two-tailed *t*-test (Sigma Plot 11.0); p < 0.05 was considered significant.
(DOCX)

S5 Table Densitometric analysis of Western blots from cerebrum at P7. Densitometric analysis of Western blots from mouse cerebra at P7 (n = 8 hypoxic and 8 normoxic mice). Calculation of p-values used Student’s unpaired, two-tailed *t*-test (Sigma Plot 11.0); p < 0.05 was considered significant.
(DOCX)

S6 Table Behavioral motor testing—Female mice. Behavioral motor testing of female mice at P21, P43 and P80. The latency to fall was analyzed in in all behavioral tests. Calculation of p-values used Student’s unpaired, two-tailed *t*-test or the Mann-Whitney Rank Sum Test in cases where the Normality of data distribution failed (Sigma Plot 11.0); p < 0.05 was considered significant.
(DOCX)
