# Supplementary figures and images for: Abbreviated Exposure to Hypoxia Is Sufficient to Induce CNS Dysmyelination, Modulate Spinal Motor Neuron Composition, and Impair Motor Development in Neonatal Mice
Source: PLoS One. 2015 May 28;10(5):e0128007. doi: 10.1371/journal.pone.0128007 (PMC4447462; doi:10.1371/journal.pone.0128007)

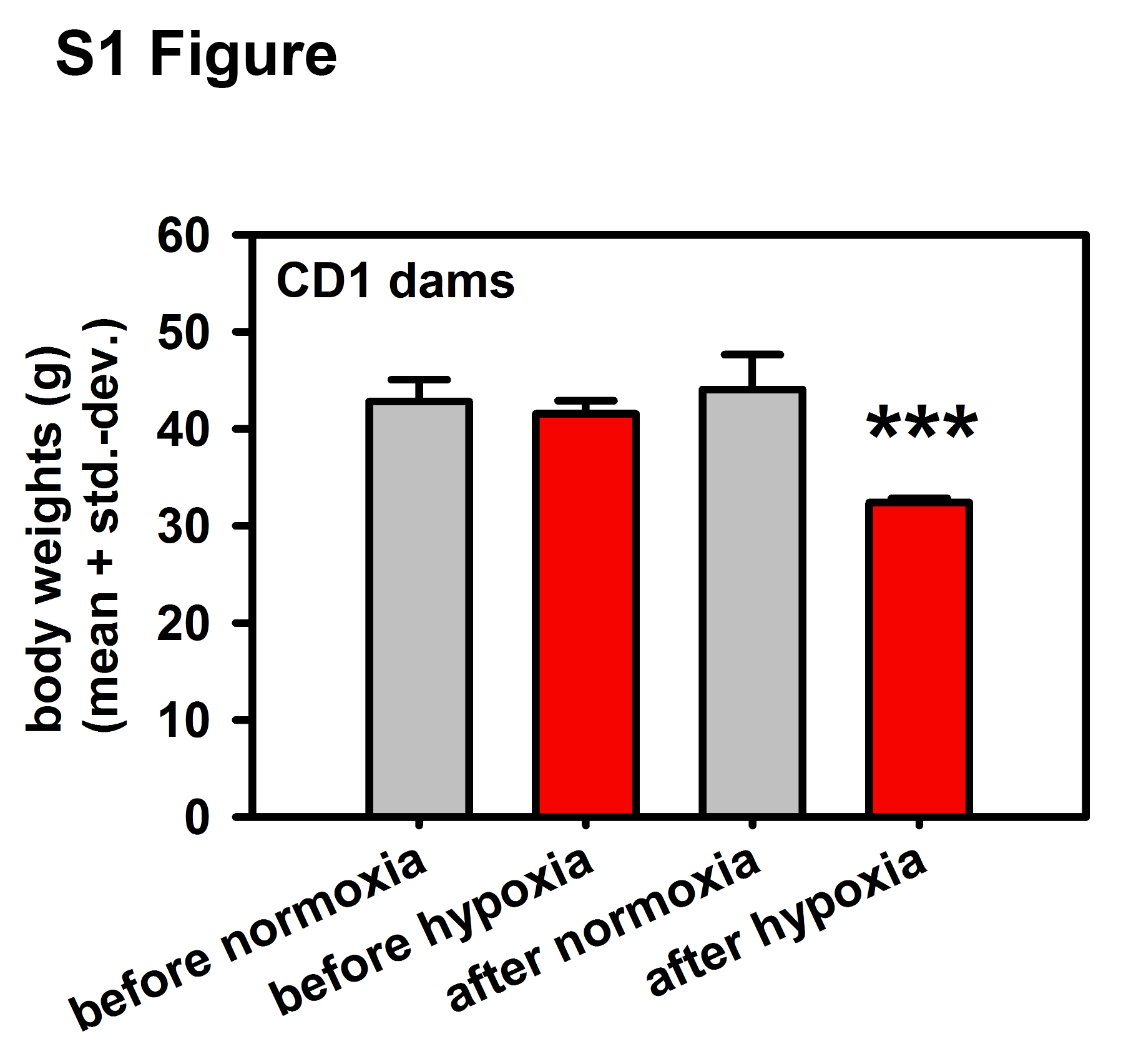

Supplement: S1 Fig — Body weights of timed pregnant CD1 dams (Charles River Laboratories) were determined right before the assignment to hypoxia or normoxia (when neonatal pups were 3 days old) and after the assignment to hypoxia or normoxia (when neonatal pups were 7 days old) (n = 12 per group). Results indicate a 25% body weight reduction in dams assigned to 4 days of hypoxia (10% O2) compared to dams in room air (normoxia) with *** equals p < 0.001; ** equals p < 0.01; * equals p < 0.05. (TIF) [file pone.0128007.s001.tif]

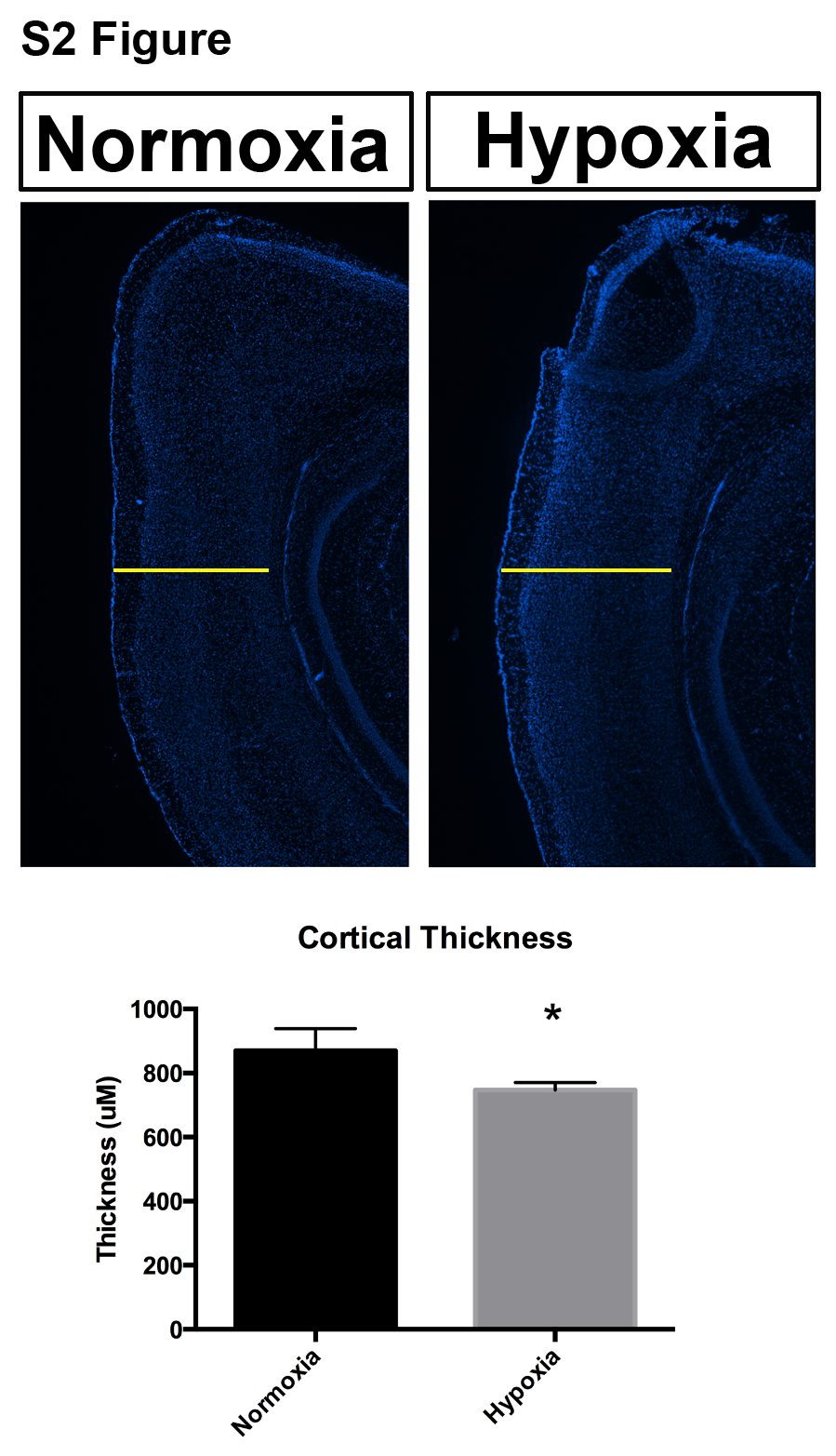

Supplement: S2 Fig — Representative cortical images from hypoxic and normoxic brains (P7) illustrating measurement of cortical thickness in level matched tissue (yellow line indicates the cortical thickness measured). Cortical thickness was determined at multiple locations per brain and quantified as mean ± std.-dev. with *** equals p < 0.001; ** equals p < 0.01; * equals p < 0.05. (TIF) [file pone.0128007.s002.tif]

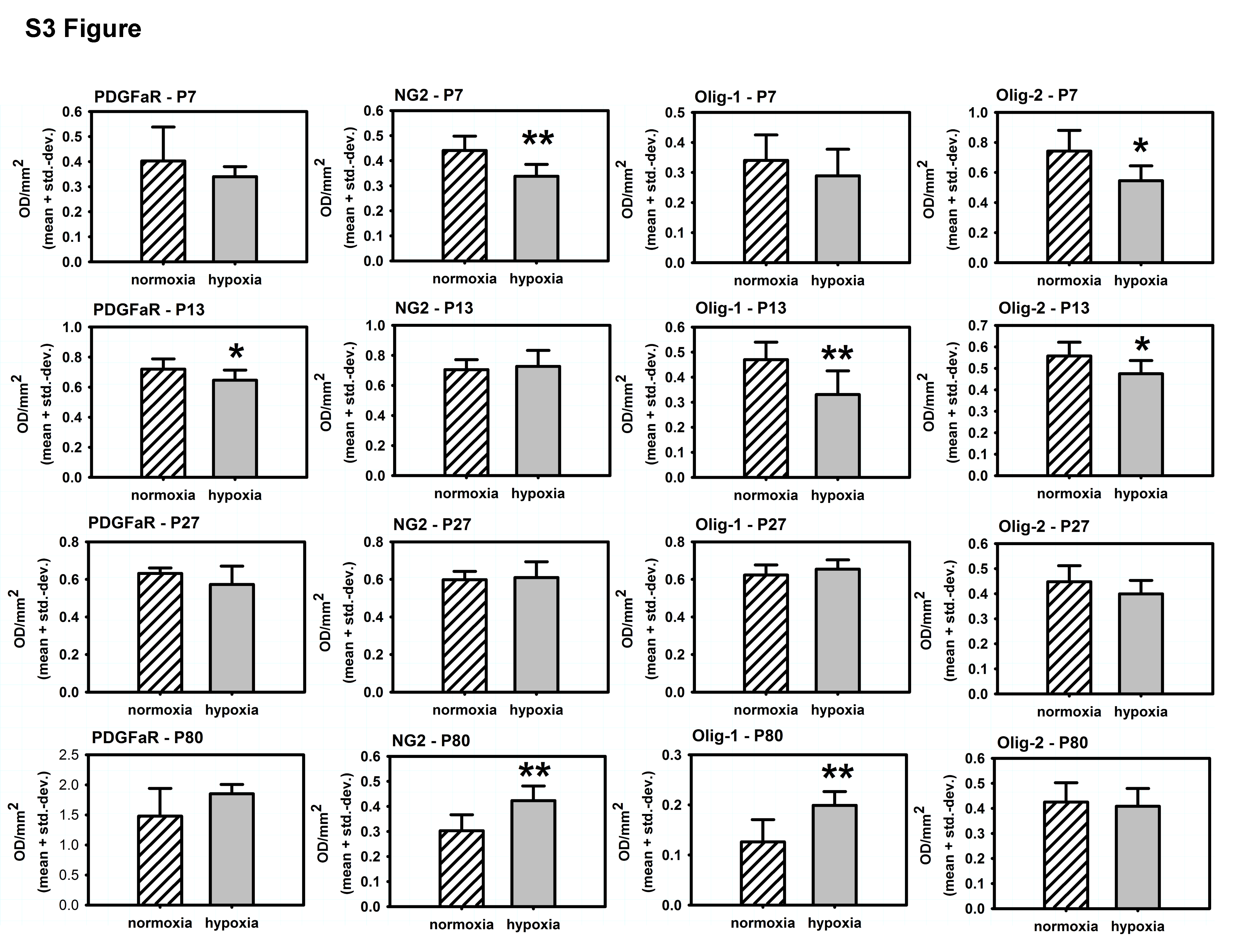

Supplement: S3 Fig — Densitometric analysis of Western blots from 3 independent experiments using total brain lysates from hypoxic and control CD1 mice at P7, P13, P27 and P80. Bar graphs show levels of OPC markers PDGFαR, NG2, Olig-2 and Olig-1. (TIF) [file pone.0128007.s003.tif]

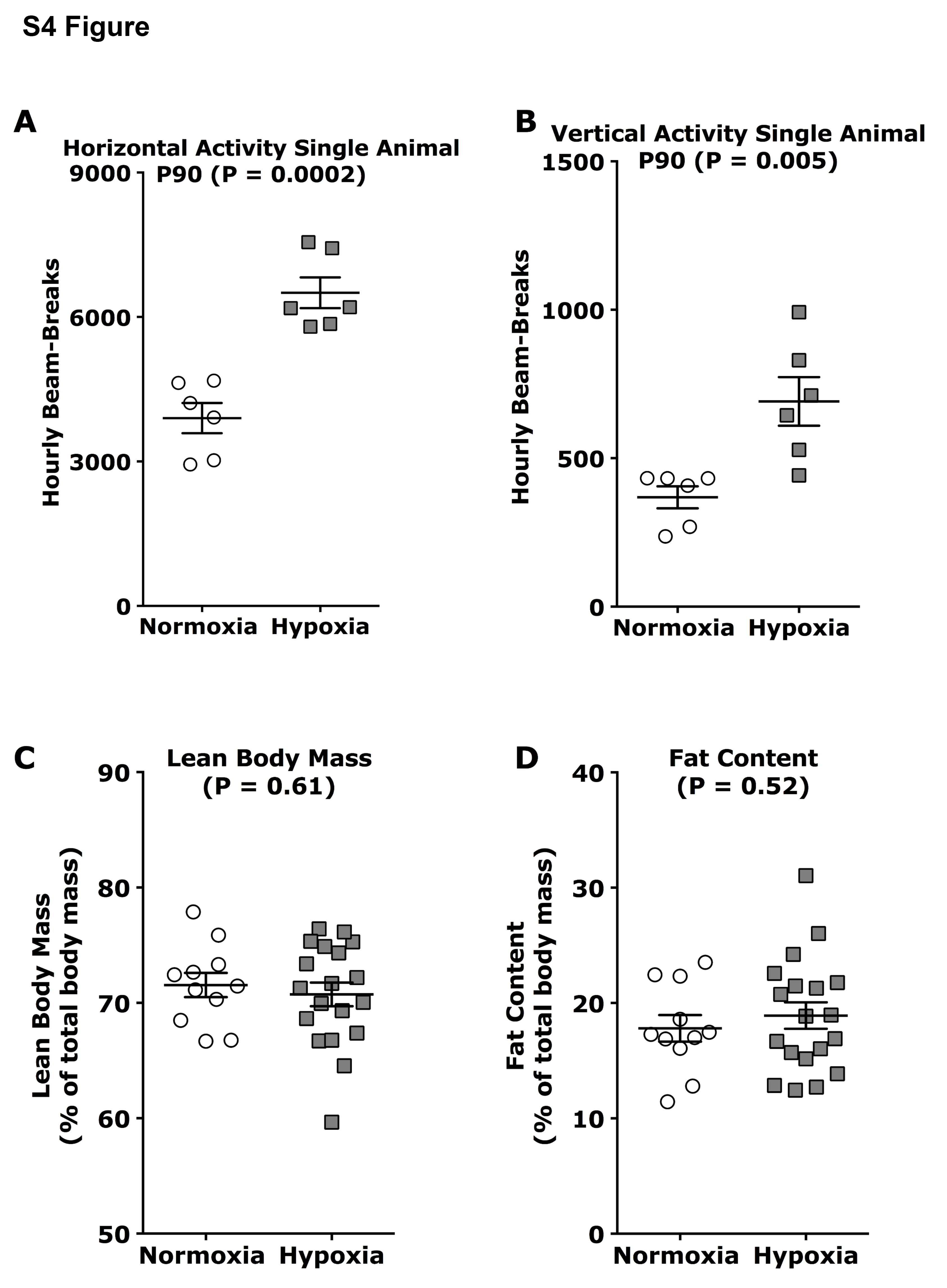

Supplement: S4 Fig — Mice reared under hypoxia (P3 → P7) or room air were tested during adulthood for global nocturnal activity (A, B) and body mass analysis (C, D). A, B: Global nocturnal activity with single mice per box (n = 6 hypoxic + 6 normoxic mice) showing horizontal hourly beambreaks (A) and vertical hourly beambreaks (B) for 5 nights from P91-P96. C, D: Echo-MRI analysis of lean body mass (C) and fat content (D) in hypoxic and control mice, *** equals p < 0.001; ** equals p < 0.01; * equals p < 0.05. (TIF) [file pone.0128007.s004.tif]
