# Supplementary material for: Abbreviated Exposure to Hypoxia Is Sufficient to Induce CNS Dysmyelination, Modulate Spinal Motor Neuron Composition, and Impair Motor Development in Neonatal Mice
Source: PLoS One. 2015 May 28;10(5):e0128007. doi: 10.1371/journal.pone.0128007 (PMC4447462; doi:10.1371/journal.pone.0128007)
Supplement: S1 Table — RT-PCR primers used to determine mouse CNS mRNA levels at P13, P27 and P80. (DOCX) [file pone.0128007.s005.docx]

**S1 Table: RT-PCR primers**

| **gene** | **primer pair** |
| --- | --- |
| PLP-1 | forward – 5’-GCTTTCCCTGGCAAGGTTTG-3’, reverse – 5’-AGCTCAGAACTTGGTGCCTC-3’ |
| MBP | forward – 5’-GGCAAGGTACCCTGGCTAAA-3’, reverse – 5’-AAATCTGCTGAGGGACAGGC-3’ |
| MOG | forward – 5’-ATCGCACTTGTGCCTACGAT-3’, reverse – 5’-GCTCCAGGAAGACACAACCA-3’ |
| β-actin | forward –5’-CCACCATGTACCCAGGCATT-3’, reverse – 5’-AGGGTGTAAAACGCAGCTCA-3’ |
