# Supplementary material for: Abbreviated Exposure to Hypoxia Is Sufficient to Induce CNS Dysmyelination, Modulate Spinal Motor Neuron Composition, and Impair Motor Development in Neonatal Mice
Source: PLoS One. 2015 May 28;10(5):e0128007. doi: 10.1371/journal.pone.0128007 (PMC4447462; doi:10.1371/journal.pone.0128007)
Supplement: S2 Table — Densitometric analysis of Western blots from mouse cerebra at P13 (n = 8 hypoxic and 8 normoxic mice). Calculation of p-values used Student’s unpaired, two-tailed t-test (Sigma Plot 11.0); p < 0.05 was considered significant. (DOCX) [file pone.0128007.s006.docx]

**S2 Table: Densitometric analysis of Western blots from cerebrum at P13**

| **CNS protein** | **10 % O2** | **21 % O2** | **Fold change rel. to control** | **p-value** |
| --- | --- | --- | --- | --- |
| CNPase | 0.29 ± 0.08 | 1.08 ± 0.14 | 0.27 | **p < 0.001** |
| PLP-1 | 0.09 ± 0.09 | 1.44 ± 0.45 | 0.06 | **p < 0.001** |
| MBP | 0.10 ± 0.10 | 1.07 ± 0.18 | 0.09 | **p < 0.001** |
| MOG | 0.77 ± 0.32 | 1.31 ± 0.13 | 0.59 | **p < 0.001** |
| PDGFRα | 0.65 ± 0.07 | 0.72 ± 0.07 | 0.90 | **p = 0.048** |
| NG2 | 0.72 ± 0.11 | 0.71 ± 0.07 | 1.03 | p = 0.627 |
| Olig-2 | 0.47 ± 0.06 | 0.56 ± 0.06 | 0.85 | **p = 0.019** |
| Olig-1 | 0.33 ± 0.09 | 0.47 ± 0.07 | 0.70 | **p = 0.005** |
| BS lectin | 0.43 ± 0.12 | 0.37 ± 0.07 | 1.15 | p = 0.300 |
