# Supplementary material for: Abbreviated Exposure to Hypoxia Is Sufficient to Induce CNS Dysmyelination, Modulate Spinal Motor Neuron Composition, and Impair Motor Development in Neonatal Mice
Source: PLoS One. 2015 May 28;10(5):e0128007. doi: 10.1371/journal.pone.0128007 (PMC4447462; doi:10.1371/journal.pone.0128007)
Supplement: S3 Table — Densitometric analysis of Western blots from mouse cerebra at P27 (n = 8 hypoxic and 8 normoxic mice). Calculation of p-values used Student’s unpaired, two-tailed t-test (Sigma Plot 11.0); p < 0.05 was considered significant. (DOCX) [file pone.0128007.s007.docx]

**S3 Table: Densitometric analysis of Western blots from cerebrum at P27**

| **CNS protein** | **10 % O2** | **21 % O2** | **Fold change rel. to control** | **p-value** |
| --- | --- | --- | --- | --- |
| CNPase | 0.25 ± 0.04 | 0.33 ± 0.05 | 0.77 | **p = 0.016** |
| PLP-1 | 0.49 ± 0.05 | 0.59 ± 0.08 | 0.83 | **p = 0.026** |
| MBP | 0.28 ± 0.02 | 0.45 ± 0.04 | 0.62 | **p < 0.001** |
| MOG | 0.30 ± 0.03 | 0.36 ± 0.05 | 0.82 | **p = 0.026** |
| PDGFRα | 0.57 ± 0.10 | 0.63 ± 0.03 | 0.91 | p = 0.310 |
| NG2 | 0.61 ± 0.08 | 0.60 ± 0.05 | 1.02 | p = 0.770 |
| Olig-2 | 0.40 ± 0.05 | 0.45 ± 0.06 | 0.89 | p = 0.187 |
| Olig-1 | 0.66 ± 0.05 | 0.62 ± 0.05 | 1.05 | p = 0.312 |
| BS lectin | 0.73 ± 0.14 | 0.30 ± 0.06 | 2.45 | **p < 0.001** |
