# Supplementary material for: Abbreviated Exposure to Hypoxia Is Sufficient to Induce CNS Dysmyelination, Modulate Spinal Motor Neuron Composition, and Impair Motor Development in Neonatal Mice
Source: PLoS One. 2015 May 28;10(5):e0128007. doi: 10.1371/journal.pone.0128007 (PMC4447462; doi:10.1371/journal.pone.0128007)
Supplement: S4 Table — Densitometric analysis of Western blots from mouse cerebra at P80 (n = 8 hypoxic and 8 normoxic mice). Calculation of p-values used Student’s unpaired, two-tailed t-test (Sigma Plot 11.0); p < 0.05 was considered significant. (DOCX) [file pone.0128007.s008.docx]

**S4 Table: Densitometric analysis of Western blots from cerebrum at P80**

| **CNS protein** | **10 % O2** | **21 % O2** | **Fold change rel. to control** | **P-value** |
| --- | --- | --- | --- | --- |
| CNPase | 1.29 ± 0.10 | 1.09 ± 0.23 | 1.18 | **p = 0.043** |
| PLP-1 | 0.38 ± 0.05 | 0.33 ± 0.07 | 1.15 | p = 0.128 |
| MBP | 0.20 ± 0.03 | 0.20 ± 0.04 | 1.04 | p = 0.602 |
| MOG | 0.37 ± 0.06 | 0.41 ± 0.05 | 0.91 | p = 0.166 |
| PDGFRα | 1.85 ± 0.16 | 1.48 ± 0.46 | 1.25 | p = 0.130 |
| NG2 | 0.42 ± 0.06 | 0.30 ± 0.06 | 1.40 | **p = 0.002** |
| Olig-2 | 0.41 ± 0.07 | 0.43 ± 0.08 | 0.96 | p = 0.661 |
| Olig-1 | 0.20 ± 0.03 | 0.13 ± 0.04 | 1.60 | **p = 0.001** |
| BS lectin | 0.66 ± 0.21 | 0.42 ± 0.24 | 1.57 | p = 0.051 |
