# Supplementary material for: Abbreviated Exposure to Hypoxia Is Sufficient to Induce CNS Dysmyelination, Modulate Spinal Motor Neuron Composition, and Impair Motor Development in Neonatal Mice
Source: PLoS One. 2015 May 28;10(5):e0128007. doi: 10.1371/journal.pone.0128007 (PMC4447462; doi:10.1371/journal.pone.0128007)
Supplement: S5 Table — Densitometric analysis of Western blots from mouse cerebra at P7 (n = 8 hypoxic and 8 normoxic mice). Calculation of p-values used Student’s unpaired, two-tailed t-test (Sigma Plot 11.0); p < 0.05 was considered significant. (DOCX) [file pone.0128007.s009.docx]

**S5 Table: Densitometric analysis of Western blots from cerebrum at P7**

| **CNS protein** | **10 % O2** | **21 % O2** | **Fold change rel. to control** | **p-value** |
| --- | --- | --- | --- | --- |
| CNPase | 0.20 ± 0.04 | 0.47 ± 0.15 | 0.43 | **p = 0.002** |
| Cleaved caspase-3 | 0.54 ± 0.07 | 0.38 ± 0.12 | 1.43 | **p < 0.022** |
| PDGFRα | 0.34 ± 0.04 | 0.40 ± 0.14 | 0.85 | p = 0.302 |
| NG2 | 0.34 ± 0.05 | 0.44 ± 0.06 | 0.77 | **p = 0.007** |
| Olig-2 | 0.55 ± 0.10 | 0.74 ± 0.14 | 0.74 | **p = 0.017** |
| Olig-1 | 0.29 ± 0.09 | 0.34 ± 0.09 | 0.85 | p = 0.054 |
| BS lectin | 0.29 ± 0.05 | 0.37 ± 0.13 | 0.79 | p = 0.184 |
