# Supplementary material for: Abbreviated Exposure to Hypoxia Is Sufficient to Induce CNS Dysmyelination, Modulate Spinal Motor Neuron Composition, and Impair Motor Development in Neonatal Mice
Source: PLoS One. 2015 May 28;10(5):e0128007. doi: 10.1371/journal.pone.0128007 (PMC4447462; doi:10.1371/journal.pone.0128007)
Supplement: S6 Table — Behavioral motor testing of female mice at P21, P43 and P80. The latency to fall was analyzed in in all behavioral tests. Calculation of p-values used Student’s unpaired, two-tailed t-test or the Mann-Whitney Rank Sum Test in cases where the Normality of data distribution failed (Sigma Plot 11.0); p < 0.05 was considered significant. (DOCX) [file pone.0128007.s010.docx]

**S6 Table: Behavioral motor testing - Female mice**

|  | **Latency to fall (s) (mean + std.-dev.)** | | |
| --- | --- | --- | --- |
| **Motor test** | **Normoxia, F** | **Hypoxia, F** | **Statistical method** |
| **P21, mesh wire** | 151.7 ± 39.6 (n = 28) | 86.5 ± 49.5 (n = 29) (p < 0.001) | Mann-Whitney Rank Sum Test |
| **P21, single wire** | 166.3 ± 26.4 (n = 22) | 130.6 ± 55.6 (n = 27) (p = 0.018) | Mann-Whitney Rank Sum Test |
| **P21, Rotarod** | 254.5 ± 65.6 (n = 17) | 169.8 ± 67.9 (n = 21) (p < 0.001) | Student’s t-test |
| **P43, mesh wire** | 152.3 ± 51.2 (n = 24) | 136.9 ± 61.6 (n = 25) (p = 0.509) | Mann-Whitney Rank Sum Test |
| **P43, single wire** | 151.5 ± 50.1 (n = 24) | 99.8 ± 77.0 (n = 21) (p < 0.015) | Mann-Whitney Rank Sum Test |
| **P43, Rotarod** | 231.5 ± 57.8 (n = 15) | 127.1 ± 61.5 (n = 23) (p < 0.001) | Mann-Whitney Rank Sum Test |
| **P80, mesh wire** | 169.6 ± 8.1 (n = 20) | 108.0 ± 18.2 (n = 14) (p = 0.002) | Student's t-test |
| **P80, single wire** | 180.0 ± 0.1 (n = 20) | 131.0 ± 19.1 (n = 14) (p = 0.004) | Student's t-test |
| **P80, Rotarod** | 190.5 ± 12.2 (n = 19) | 207.0 ± 14.3 (n = 20) (p = 0.3890) | Student's t-test |
